# Phenotypic changes of γδ T cells in *Plasmodium falciparum* placental malaria and pregnancy outcomes in women at delivery in Cameroon

**DOI:** 10.3389/fimmu.2024.1385380

**Published:** 2024-05-17

**Authors:** Chris Marco Mbianda Nana, Bodin Darcisse Kwanou Tchakounté, Bernard Marie Zambo Bitye, Balotin Fogang, Berenice Kenfack Tekougang Zangue, Reine Medouen Ndeumou Seumko’o, Benderli Christine Nana, Rose Gana Fomban Leke, Jean Claude Djontu, Rafael José Argüello, Lawrence Ayong, Rosette Megnekou

**Affiliations:** ^1^ Department of Animal Biology and Physiology, Faculty of Sciences, University of Yaoundé I, Yaoundé, Cameroon; ^2^ Immunology Laboratory of the Biotechnology Center, University of Yaoundé I, Yaoundé, Cameroon; ^3^ Malaria Research Unit, Centre Pasteur du Cameroun, Yaoundé, Cameroon; ^4^ CNRS, INSERM, CIML, Centre d’Immunologie de Marseille, Aix-Marseille University, Marseille, France

**Keywords:** *Plasmodium falciparum*, placental malaria, γδ T cells, HLA-DR, TIM-3, PD1, pregnancy outcomes, women

## Abstract

**Introduction:**

Depending on the microenvironment, γδ T cells may assume characteristics similar to those of Th1, Th2, Th17, regulatory T cells or antigen presenting cells. Despite the wide documentation of the effect of Th1/Th2 balance on pregnancy associated malaria and outcomes, there are no reports on the relationship between γδ T cell phenotype change and Placental Malaria (PM) with pregnancy outcomes. This study sought to investigate the involvement of γδ T cells and its subsets in placental *Plasmodium falciparum* malaria.

**Methods:**

In a case-control study conducted in Yaoundé, Cameroon from March 2022 to May 2023, peripheral, placental and cord blood samples were collected from 50 women at delivery (29 PM negative: PM- and 21 PM positive: PM+; as diagnosed by light microscopy). Hemoglobin levels were measured using hemoglobinometer. PBMCs, IVBMCs and CBMCs were isolated using histopaque-1077 and used to characterize total γδ T cell populations and subsets (Vδ1^+^, Vδ2^+^, Vδ1^-^Vδ2^-^) by flow cytometry.

**Results:**

Placental *Plasmodium falciparum* infection was associated with significant increase in the frequency of total γδ T cells in IVBMC and of the Vδ1^+^ subset in PBMC and IVBMC, but decreased frequency of the Vδ2^+^ subset in PBMC and IVBMC. The expression of the activation marker: HLA-DR, and the exhaustion markers (PD1 and TIM3) within total γδ T cells and subsets were significantly up-regulated in PM+ compared to PM- group. The frequency of total γδ T cells in IVBMC, TIM-3 expression within total γδ T cells and subsets in IVBMC, as well as HLA-DR expression within total γδ T cells and Vδ2^+^ subset in IVBMC were negatively associated with maternal hemoglobin levels. Furthermore, the frequency of total γδ T cells in PBMC and PD1 expression within the Vδ2^+^ subset in CBMC were negatively associated with birth weight contrary to the frequency of Vδ1^-^Vδ2^-^ subset in PBMC and HLA-DR expression within the Vδ2^+^ subset in IVBMC which positively associated with maternal hemoglobin level and birth weight, respectively.

**Conclusion:**

The data indicate up-regulation of activated and exhausted γδ T cells in *Plasmodium falciparum* placental malaria, with effects on pregnancy outcomes including maternal hemoglobin level and birth weight.

## Introduction

Pregnancy-associated malaria remains a major global health problem. Each year, more than 12.7 million women in the WHO African Region are exposed to malaria infection during pregnancy (1). Poor pregnancy outcomes such as maternal anemia, preterm delivery, low birth weight, and stillbirth (2, 3) are caused by placental malaria, characterized by the sequestration of infected red blood cells (IRBC) in the placental tissue. This condition is mediated by the VAR2CSA antigen expressed on IRBC surface which specifically bind to receptors called Chondroitin Sulfate A (CSA) on the placental syncytiotrophoblast lining the intervillous space of the placenta (4, 5). The sequestration of IRBC in the placental tissue is associated with altered immune responses due to increased stimulation of parasitic antigens (6). Indeed, during the parasite replication cycle, infected red blood cells at the schizont stage release several antigens including phosphoantigens, the non-peptidic intermediate metabolites of the isoprenoid production pathway: (E)-4-hydroxy-3-methyl-but-2-enyl pyrophosphate (HMBPP) produced by the 1-deoxy-D-xylulose-5-phosphate (DOXP) pathway and isopentenyl-pyrophosphate (IPP) from both the DOXP and mevalonate pathways (7). These phosphoantigens can be recognized directly by TCRγδs leading to the activation and proliferation of γδ T cells (7).

γδ T cells are a unique subpopulation of double-negative T cells (CD4^-^CD8^-^), known to be rare in secondary lymphoid organs but enriched in many peripheral tissues such as intestine, skin, and lung (8). They exhibit characteristics of both innate and adaptive immune cells (9). In adult peripheral blood, the most abundant subset of γδ T cells expresses the Vγ9^+^ and Vδ2^+^ T cell receptor chains (also called Vγ2^+^Vδ2^+^ or Vδ2^+^), representing 1–10% of total T cells (10) and 50–95% of the γδ T cells in the blood (11, 12). During acute malaria infection, Vγ9Vδ2 T cells are rapidly activated reaching proportions of up to 30% of circulating T cells (13, 14). In infected individuals, the Vδ2^+^ subset has been shown to inhibit Plasmodium falciparum blood stage development through various soluble cytotoxic mediators (IFNγ, TNFα). Other reports suggest that these cells may play a dual role by promoting cerebral malaria pathology and contributing to control parasite density (12, 15, 16). However, some studies conducted in children living in endemic areas with high malaria transmission report a loss and dysfunction of the Vδ2^+^ subset due to expression of the PD1 and TIM-3 checkpoint proteins that are associated with clinical tolerance to malaria (15) in favor of the Vδ1^+^ subset (17).

The Vδ1^+^ subset resides primarily in tissues and organs such as thymus, spleen, liver, genital tract, and intestinal epithelium (18, 19). It generally constitutes a minority (≤ 20%) of the γδ T cells in adult peripheral blood. However, more and more recent studies show that this subset can also circulate in peripheral blood, although the mechanisms remain unclear. In HIV-infected patients, the proportion of the Vδ1^+^ subset in the peripheral blood was higher compared to that of Vδ2^+^ (20). Although the mechanisms of recognition of Plasmodium falciparum-infected red blood cells by these T cell subsets remain poorly understood, it has been reported that the proportion of the Vδ1^+^ subset dominated *ex-vivo* in exposed healthy individuals living in areas of stable malaria transmission (17).

The role of γδ T cells in women during pregnancy remains controversial. Some studies have shown that early pregnancy and peri-implantation were associated with an increased amount of these cells in the third trimester compared to the early first trimester (21). Furthermore, other studies have reported an association between a decrease of peripheral γδ T cells and recurrent spontaneous abortion (22). Conversely, data from recent studies associate recurrent spontaneous abortion with an increased proportion of peripheral γδ T cells and Vδ2^+^ subset (23, 24). Nevertheless it has been recognized that γδ T cells may assume characteristics similar to those of Th1, Th2, Th17, regulatory T cells and antigen presenting cells depending on the microenvironment (25, 26). This dichotomy of γδ T cells and the Vδ2^+^ and Vδ1^+^ subsets make it difficult to assess their role during pregnancy, especially in pregnant women with placental *Plasmodium falciparum* malaria. The dichotomous function can be characterized by the expression of the immunoregulatory markers PD1 or TIM-3, which can control the cytotoxic function of T cells. Indeed, studies have shown that co-expression of Tim-3 and PD1 on CD8^+^ T cells is associated with a decrease in the cytolytic activity of this lymphocyte and the development of a Th2 dominant milieu in the decidua, essential for the maintenance of a normal pregnancy (27). However, in women with recurrent spontaneous abortions, TIM-3 expression was significantly decreased in CD8^+^ T cells, whereas PD1 expression was up-regulated in CD4^+^ T cells (28). The present study aims to examine the phenotypic changes in γδ T cells and their subsets including the expression of activation (HLA-DR) and exhaustion (TIM-3 and PD1) biomarkers in the context of *Plasmodium falciparum* placental infection in matched peripheral, placental and cord blood, and to correlate their expression with pregnancy outcomes. This study may help improving the understanding of the role play by γδ T cells on the immunopathophysiology of placental malaria and pregnancy outcomes, thus contributing towards development of new therapeutic strategies.

## Methods

### Study design and sample collection

This case-control (2:3 ratio) study was carried out from March 2022 to May 2023. Study participants were pregnant women living in the urban and sub-urban areas of Yaoundé, Cameroon, who attended the health centers for delivery. A total of 50 peripheral, placental, and cord whole blood were collected from each participant (21 placental malaria positive and 29 negative) at delivery at the Marie Reine Health Center of Etoudi and at the Catholic Hospital of Marie des Anges of Koabang. The two healthcare centers are located in two outlying districts of Yaoundé where malaria transmission is perennial, with 2 wet and 2 dry seasons. Information on the mother’s age, mother’s health, gravidity, parity, gestational age, use of the intermittent preventive treatment with sulfadoxine-pyrimethamine during pregnancy (IPTp-SP), use of insecticide-treated bed nets (ITN), and baby’s weight at delivery were recorded in a standard questionnaire. The participants with pre-existing health conditions (e.g., preeclampsia, diabetes, toxoplasmosis, hepatitis, syphilis and HIV positive results) were excluded from the study. Birth weights lower than 2.5 kg were considered low as per the World Health Organization’s guidelines (29). Maternal venous, placental and cord blood samples were aseptically collected at delivery in BD Vacutainer heparinized tubes. A fragment of placental tissue was collected from the maternal side of the placenta immediately following delivery and used to diagnose placental malaria (PM). Blood samples were processed on the day of collection.

### Diagnosis of placental malaria and determination of hemoglobin levels

Thick blood smears from maternal, placental and cord blood samples, and impression smears from placental tissue were stained using Giemsa and examined by at least two skilled microscopists for the presence of malaria parasites. *Plasmodium falciparum* infection was further confirmed using the Commercialized One Step HRP-II and pLDH RDT rapid diagnostic test (SD Bioline malaria antigen P.f/Pan, Standard Diagnostics Inc, Kyonggi-do, Korea). Participants were considered placental malaria positive if infected erythrocytes were detected in impression smears of placenta tissue and/or placental or peripheral blood smears. Hemoglobin (Hb) levels in maternal and neonate cord blood were determined using a hemoglobinometer (ACON Laboratories, INC, San Diego, USA). Participants were considered anemic if Hb < 11 g/dl whereas neonates were considered anemic if Hb < 12,5 g/dl (30).

### Isolation of PBMCs, IVBMCs and CBMCs

Isolated cells consist of Peripheral Blood Mononuclear Cells (PBMC) from the mother, Intervillous Blood Mononuclear Cells (IVBMC) from the placental and Cord Blood Mononuclear Cells (CBMC) from the cord. Briefly, approximately 15 ml of freshly collected peripheral, placental or cord blood samples were each diluted 1:1 in 1X PBS (without Ca^2+^ and Mg^2+^) supplemented with 2% FBS (Thermo Fisher Scientific, USA) and then layered onto 15ml Histopaque 1077 (Sigma-Aldrich, USA) in a 50 ml Falcon tube. Tubes were centrifuged for 30 minutes at 800g and 20°C without brake. The PBMCs, IVBMCs and CBMCs layers were recovered, washed twice with 1X PBS (without Ca^2+^ and Mg^2+^) + 2% FBS by centrifugation at 600 g and 20°C for 8 minutes. The viability of the recovered cells was determined by Trypan blue dye exclusion method. Only samples with cell viability greater than 95% were used in the assay. Approximately 1×10^6^ of isolated PBMCs, IVBMCs or CBMCs were re-suspended in 1mL of 1X PBS for surface staining.

### Surface staining

The PBMCs, IVBMCs and CBMCs staining were done in 96-well Microplate V shaped bottom. The cells were washed and re-suspended in 50 µl of FACS buffer (1X PBS without Ca^2+^ and Mg^2+^, 5% FBS, 5mM EDTA) with human FcR Blocking Reagent (Miltenyi Biotech, USA) and incubated for 10 min at 4°C in the dark. After washing, the pellet was re-suspended in 50 µl of antibody staining mix and incubated for 30 min at 4°C in the dark. Cells were then washed with 200 µl of FACS buffer at 1800 rpm for 3 min and re-suspended in FACS buffer prior to acquisition. The following monoclonal antibodies were used for surface staining: Brilliant Violet (BV) 510 labelled anti-CD3 (clone UCHT1) (BD Biosciences, USA); Phycoerythrin (PE) labelled anti-TCRγδ PE (clone B1); Allophycocyanin-Cyanine 7 (APC-Cy7) labelled anti-TCR V delta 2 (clone B6), BV421 labelled anti-TIM-3 (clone F38–2E2), APC labelled anti-PD1 (clone EH12.2H7) (Biolegend, USA); PE-Cy7 labelled anti-TCR V delta 1 (clone TS8.2) and Fluorescein Isothiocyanate (FITC) labelled anti-HLA-DR (clone L243) (ThermoFisher Scientific, USA). Samples were acquired within 2 hours of staining on a BD FACS Canto II flow cytometer equipped with a BD FACSDiva software version 6.1.3 (Becton Dickinson, USA).

### Flow cytometry data analysis

Flow cytometry data were analyzed using FlowJo software version 10.8.1 (Tree Star, Inc) and OMIQ. Color compensation was performed using beads stained for each fluorochrome. Only samples from which at least 100 000 CD3^+^ single cells were acquired were included in the analysis. A lymphocyte gate was set based on SSC-A/FSC-A, followed by single cell gating based on FSC-H/FSC-A, and CD3^+^ events within this gate analyzed. Unsupervised flow cytometry analysis of TCRγδ^+^ cells in PBMC, IVBMC and CBMC samples were done using Uniform Manifold Approximation Projection (UMAP) along with the FlowSOM automated clustering tool of OMIQ. 5000 TCRγδ^+^ cells for each study were concatenated and dimensionality reduction was assessed using the UMAP (15 nearest neighbors, 0.4 minimum distance). For clustering visualization, we used FlowSOM (6 metaclusters). Markers considered in the analyses included TCR Vδ1, TCR Vδ2, HLA-DR, PD1 and TIM-3.

### Statistical analysis

Statistical analyses were performed using GraphPad Prism version 9.4.1. Results were reported as means with standard deviation or medians with interquartile ranges. Comparisons between two groups were assessed using Mann-Whitney rank sum test. Spearman rank order correlation coefficient (r_s_) was used to evaluate associations. Proportions were compared using Fisher’s exact test. P values <0.05 were considered statistically significant.

## Results

### Study population

Samples of 29 women without placental malaria (PM-) and 21 women with placental malaria (PM+) were used in the present study. The basic characteristics of the study population are summarized in **Table 1**. Mean age, median parity and median gravidity of PM- women were not significantly different from those of PM+ women (p=0.343; p=0.086 and p=0.535 respectively). Median maternal hemoglobin level was significantly lower (p=0.002) in PM+ women (10.67 g/dL) compared to PM- women (12.67 g/dL). Moreover, the prevalence of maternal anemia was significantly higher (p=0.003) in PM+ women (52.38%) compared to PM- women (10.71%). Median gestational age was not significantly different between PM- and PM+ women (p= 0.212). No significant difference (p=0.186) was observed in fetal hemoglobin level between PM+ women (15.66 g/dL) and PM- women (14.50 g/dL). A similar trend was observed with the prevalence of fetal anemia: 50% in PM- women and 38.10%) in PM+ women (p=0.563). Baby weight at delivery was significantly lower for PM+ women (3,045g) compared to PM- women (3,353g) (p=0.014). In terms of proportion of women who took IPTp-SP or used ITNs during pregnancy, no significant difference (p>0.999 and p=0.683, respectively) was observed between PM- women (88.89% and 82.14), and PM+ women (85.71% and 90.48%).

**Table 1:** General characteristics of the study population.

Variables	PM- women n=29	PM+ women n=21	p values
Age in years (mean ± SD)	27.03 ± 5.402	25.65 ± 4.801	0.343
Parity [median and 25% - 75% IQR]	2 [1 – 4]	2 [1 - 2.5]	0.086
Gravidity [median and 25% - 75% IQR]	3 [2 – 4]	2 [1 – 4]	0.535
Maternal hemoglobin levels in g/dL, median [25% - 75% IQR]	12.67 [11.66 - 14.25]	10.67 [10.33 - 12.33]	0.002
Percentage of maternal anemia (%)	3/28 (10.71)	11/21 (52.38)	0.003
Gestational age,median [25% - 75% IQR]	39 [38 – 41]	39 [37.25 - 40]	0.212
Fetal hemoglobin levels in g/dL (mean ± SD)	14.50 ± 2.01	15.66 ± 1.90	0.221
Percentage of fetal anemia (%)	14/28 (50)	8/21 (38.10)	0,563
Baby birth weight (mean g ± SD)	3353 ± 397.9	3045 ± 413.1	0.014
IPTp-SP usage (%)	24/27 (88.89)	18/21 (85.71)	>0.999
ITNs usage (%)	23/28 (82.14)	19/21 (90.48)	0.683

PM-, Placental Malaria negative women; PM+, Placental Malaria positive women; IPTp-SP, Intermittent preventive treatment with sulphadoxine-pyrimethamine; ITNs, Insecticide treated bed net; IQR, Interquartile ranges; %, Percentage; SD, Standard deviation.

### Association of *P. falciparum* placental infection with increased frequency of total γδ T cells and Vδ1^+^ subset and decreased frequency of Vδ2^+^ subset in PBMC and IVBMC

**Figure 1:**
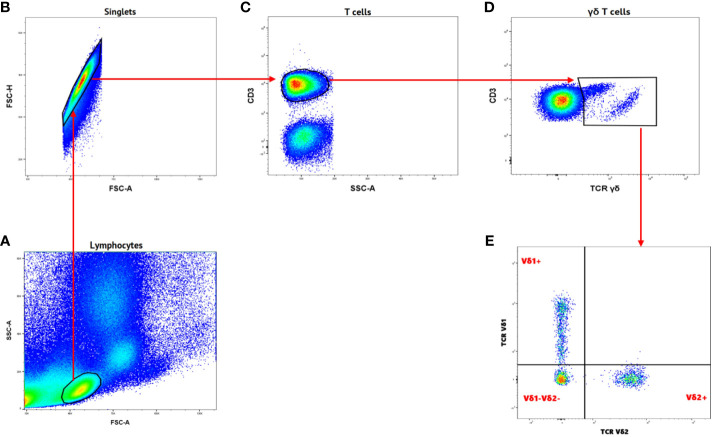
Gating strategy of Gamma Delta T cells and different subsets. **(A)** Samples were initially gated on lymphocytes based on SSC-A/FSC-A gating. **(B)** Single cells were gated from lymphocytes using FSC-H/FSC-A. **(C)** T cells were gated from singlets as CD3^+^ cells. **(D)** Gamma delta T cells were gated from CD3^+^ as TCRγδ^+^. **(E)** Different subsets of Gamma delta T cells were characterized TCR Vδ1 and TCR Vδ2 markers as Vδ1^+^, Vδ2^+^ and Vδ1^-^Vδ2^-^.

**Figure 2:**
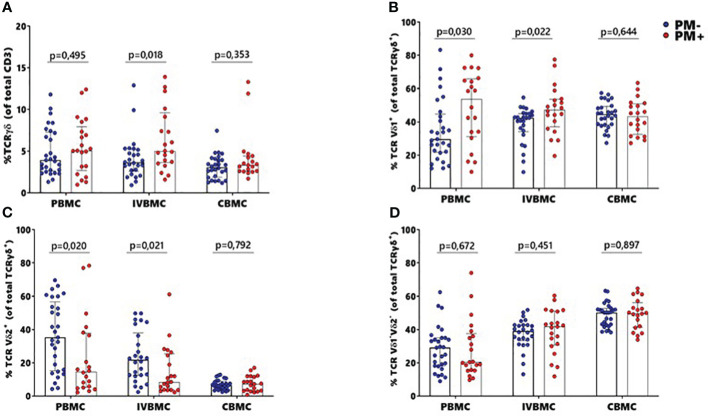
Frequencies of γδ T cells and different subsets Vδ1^+^, Vδ2^+^, Vδ1^-^Vδ2^-^ in women at delivery. γδ T cells and different subsets were analyzed through multiparametric flow cytometry in PBMC, IVBMC and CBMC of PM- (in blue) and PM+ (in red) women. **(A–D)** frequency of **(A)** total TCR γδ^+^ **(B)** TCR Vδ1^+^ **(C)** TCR Vδ2^+^ and **(D)** TCR Vδ1^-^Vδ2^-^. Frequencies were compared in each group using Mann-Whitney test. PM-, Placental Malaria negative women; PM+, Placental Malaria positive women; PBMC, Peripheral blood mononuclear cell; IVBMC, Intervillous blood mononuclear cell; CBMC, Cord blood mononuclear cell. Each dot represents a single individual.

**Figure 3:**
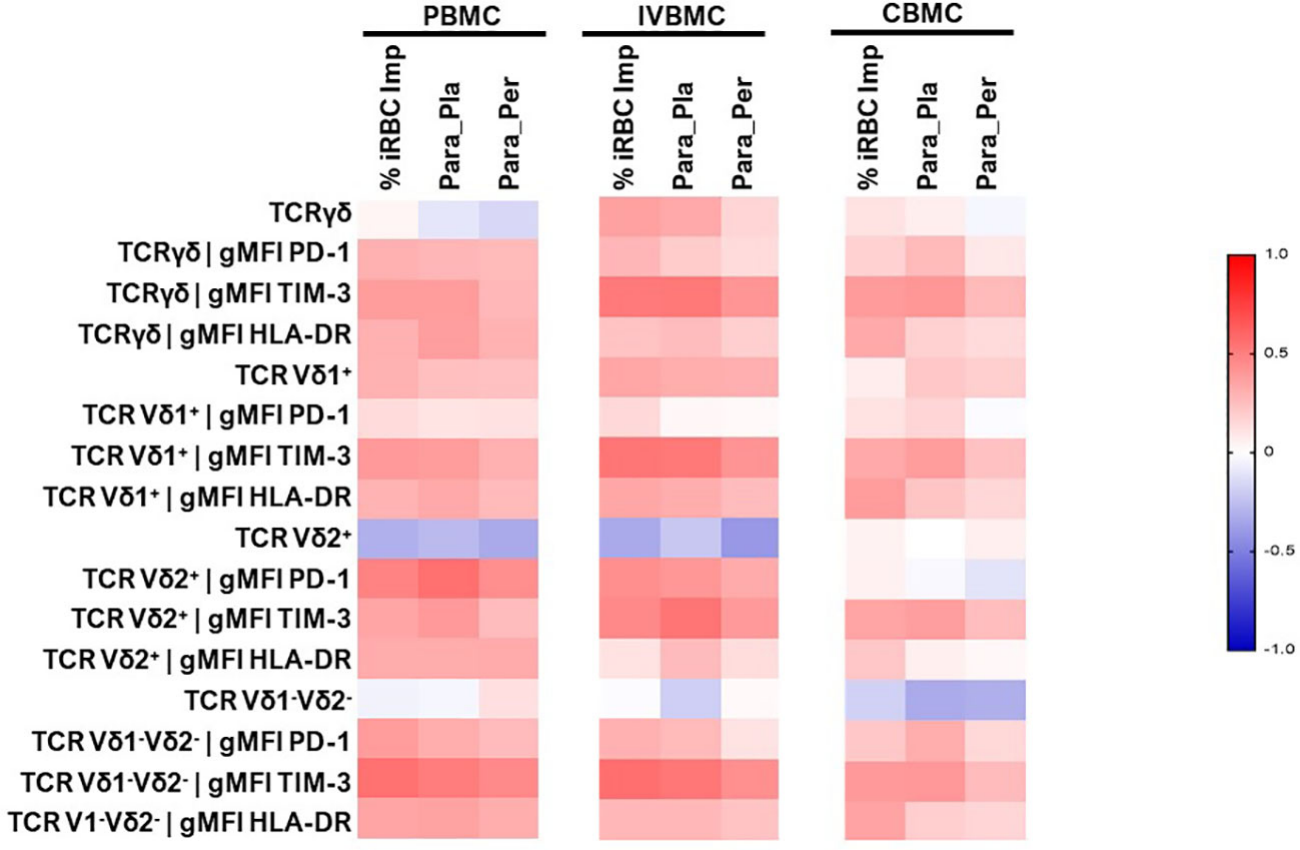
Heatmap representing the correlation between the γδ T cells phenotype and different parasitemias. The correlation between γδ T cells profile in PBMC, IVBMC, CBMC and different parasitemias was determined using Sperman’s rank Order correlation test. %iRBC Imp, parasitemias from placenta tissue impression smear; Para_Per, Peripheral blood parasitemias; Para_Pla, Placental blood parasitemias; PBMC, Peripheral blood mononuclear cell; IVBMC, Intervillous blood mononuclear cell; CBMC, Cord blood mononuclear cell.

γδ T cells and its subsets were analyzed using the gating strategy shown in **Figure 1** and **Supplementary Figure 1**. The frequency of total γδ T cells, Vδ1^+^, Vδ2^+^ and Vδ1^-^Vδ2^-^ subsets in PBMC, IVBMC, and CBMC between PM- and PM+ women are presented in (**Figure 2**) and the correlations in (**Figure 3**; **Tables 2**–**4**). We observed no significant difference in proportions of cells expressing each marker type, comparing the infected and uninfected samples, reason for which only gMFIs data were presented in the manuscript (**Tables 2**–**4**). The frequency of total γδ T cells in IVBMC and of the Vδ1^+^ subset in PBMC and IVBMC were significantly higher in PM+ women compared to PM- women (p=0.018, p=0.030 and p=0.022, respectively) (**Figures 2A, B**). Although the frequency of total γδ T cells in PBMC was high in PM+ women compared to PM- women, the difference was not significant (p=0.495). Furthermore, the frequency of total γδ T cells in IVBMC correlated positively with parasitemia from placenta tissue impression smear and placental blood parasitemia [(r_s_ =0.366; p=0.011) and (r_s_ =0.330; p=0.023, respectively)] (**Figure 3** and **Table 3**). Indeed, the frequency of the Vδ1^+^ subset correlated positively with parasitemia from placenta tissue impression smears in PBMC (r_s_ =0.300; p=0.036) and with parasitemia from placenta tissue impression smears, peripheral and placental blood parasitemia in IVBMC (0.313 ≤ r_s_ ≤ 0.347; 0.017 ≤ p ≤ 0.034) (**Figure 3**; **Tables 2**, **3**). In contrast, the frequency of the Vδ2^+^ subset in PBMC and IVBMC were significantly lower in PM+ compared to PM- women (p=0.020 and p=0.021, respectively) (**Figure 2C**). In addition, the frequency of the Vδ2^+^ subset in PBMC, IVBMC correlated negatively with parasitemia from placenta tissue impression smear and peripheral blood parasitemia (-0.404 ≤ r_s_ ≤ -0.313; 0.005 ≤ p ≤ 0.028) (**Figure 3**; **Tables 2**, **3**)., suggesting that *P. falciparum* placental infection leads to a selective loss of Vδ2^+^ subset. A similar trend in the Vδ1^-^Vδ2^-^ subset in PBMC was observed between the two groups of women although not significant (p=0.672) (**Figure 2D**). Regarding the frequency of total γδ T cells, Vδ1^+^, Vδ2^+^ and Vδ1^-^Vδ2^-^ subsets in CBMC, no significant difference was observed between PM+ and PM- women (p=0.353; p=0.644; p=0.792 and p= 0.897, respectively) (**Figures 2A–D**, **3**, **Table 4**). Together, these data indicate that they are significant differences in the expansion of Vδ1^+^ and Vδ2^+^ subsets in PBMC and IVBMC during *P. falciparum* placental infection.

**Table 2:** Correlations between γδ T cells phenotype in PBMC and different parasitemias.

PBMC		%iRBCs Imp	Para_Pla	Para_Per
**TCRγδ^+^**	r_s_	0.038	-0.099	-0.153
	p value	0.792	0.494	0.289
**TCRγδ^+^ | gMFI PD1**	r_s_	**0.305**	**0.283**	0.269
	p value	**0.031**	**0.046**	0.059
**TCRγδ^+^ | gMFI TIM-3**	r_s_	**0.383**	**0.387**	0.278
	p value	**0.007**	**0.006**	0.054
**TCRγδ^+^ | gMFI HLA-DR**	r_s_	**0.306**	**0.377**	**0.299**
	p value	**0.033**	**0.008**	**0.037**
**TCR Vδ1^+^**	r_s_	**0.300**	0.248	0.240
	p value	**0.036**	0.086	0.097
**TCR Vδ1^+^ | gMFI PD1**	r_s_	0.133	0.106	0.116
	p value	0.355	0.464	0.421
**TCR Vδ1^+^ | gMFI TIM-3**	r_s_	**0.393**	**0.384**	**0.308**
	p value	**0.005**	**0.006**	**0.031**
**TCR Vδ1^+^ |gMFI HLA-DR**	r_s_	**0.295**	**0.330**	0.270
	p value	**0.039**	**0.021**	0.061
**TCR Vδ2^+^**	r_s_	**-0.313**	-0.273	**-0.337**
	p value	**0.028**	0.058	**0.018**
**TCR Vδ2^+^ | gMFI PD1**	r_s_	**0.485**	**0.560**	**0.441**
	p value	**0.0003**	**0.00002**	**0.001**
**TCR Vδ2^+^ | gMFI TIM-3**	r_s_	**0.353**	**0.394**	0.262
	p value	**0.013**	**0.005**	0.069
**TCR Vδ2^+^ |gMFI HLA-DR**	r_s_	**0.322**	**0.322**	**0.327**
	p value	**0.024**	**0.024**	**0.022**
**TCR Vδ1^-^Vδ2^-^**	r_s_	-0.052	-0.038	0.125
	p value	0.718	0.792	0.388
**TCR Vδ1^-^Vδ2^-^ | gMFI PD1**	r_s_	**0.387**	**0.319**	0.264
	p value	**0.006**	**0.024**	0.064
**TCR Vδ1^-^Vδ2^-^ | gMFI TIM-3**	r_s_	**0.551**	**0.509**	**0.456**
	p value	**0.00004**	**0.0002**	**0.001**
**TCR Vδ1^-^Vδ2^-^ |gMFI HLA-DR**	r_s_	**0.353**	**0.362**	**0.318**
	p value	**0.013**	**0.011**	**0.026**

PBMC, Peripheral blood mononuclear cell; %iRBC Imp, % iRBCs of impression smear (from placenta tissue); Para Per, Peripheral blood parasitemia; Para Pla, Placental blood parasitemia. r_s_, coefficient of correlation; p value, coefficient of significance.

Bold values highlight a p-value less than 0.05.

**Table 3:** Correlations between γδ T cells phenotype in IVBMC and different parasitemias.

IVBMC		%iRBCs Imp	Para_Pla	Para Per
**TCRγδ^+^**	r_s_	**0.366**	**0.330**	0.161
	p value	**0.011**	**0.023**	0.284
**TCRγδ^+^ | gMFI PD1**	r_s_	0.283	0.202	0.136
	p value	0.054	0.173	0.369
**TCRγδ^+^ | gMFI TIM-3**	r_s_	**0.519**	**0.522**	**0.408**
	p value	**0.0002**	**0.0002**	**0.005**
**TCRγδ^+^ | gMFI HLA-DR**	r_s_	0.234	0.260	0.188
	p value	0.117	0.081	0.217
**TCR Vδ1^+^**	r_s_	**0.347**	**0.318**	**0.313**
	p value	**0.017**	**0.029**	**0.034**
**TCR Vδ1^+^ | gMFI PD1**	r_s_	0.148	0.028	0.021
	p value	0.322	0.854	0.890
**TCR Vδ1^+^ | gMFI TIM-3**	r_s_	**0.536**	**0.522**	**0.419**
	p value	**0.0001**	**0.0002**	**0.004**
**TCR Vδ1^+^ |gMFI HLA-DR**	r_s_	**0.344**	**0.318**	0.260
	p value	**0.019**	**0.031**	0.084
**TCR Vδ2^+^**	r_s_	**-0.334**	-0.223	**-0.404**
	p value	**0.022**	0.132	**0.005**
**TCR Vδ2^+^ | gMFI PD1**	r_s_	**0.437**	**0.406**	**0.329**
	p value	**0.002**	**0.005**	**0.026**
**TCR Vδ2^+^ | gMFI TIM-3**	r_s_	**0.459**	**0.541**	**0.395**
	p value	**0.001**	**0.0001**	**0.007**
**TCR Vδ2^+^ |gMFI HLA-DR**	r_s_	0.110	0.264	0.131
	p value	0.467	0.076	0.391
**TCR Vδ1^-^Vδ2^-^**	r_s_	-0.014	-0.190	0.017
	p value	0.924	0.200	0.911
**TCR Vδ1^-^Vδ2^-^ | gMFI PD1**	r_s_	**0.305**	0.269	0.107
	p value	**0.037**	0.067	0.479
**TCR Vδ1^-^Vδ2^-^ | gMFI TIM-3**	r_s_	**0.561**	**0.531**	**0.435**
	p value	**0.00005**	**0.0001**	**0.003**
**TCR Vδ1^-^Vδ2^-^ |gMFI HLA-DR**	r_s_	0.280	0.278	0.232
	p value	0.060	0.061	0.125

IVBMC, Intervillous blood mononuclear cell; %iRBC Imp, % iRBCs of impression smear (from placenta tissue); Para Per, Peripheral blood parasitemia; Para Pla, Placental blood parasitemia; r_s_, coefficient of correlation; p value, coefficient of significance.

Bold values highlight a p-value less than 0.05.

**Table 4:** Correlations between γδ T cells phenotype in CBMC and different parasitemias.

CBMC		%iRBCs Imp	Para_Pla	Para Per
**TCRγδ^+^**	r_s_	0.109	0.064	-0.036
	p value	0.461	0.668	0.808
**TCRγδ^+^ | gMFI PD1**	r_s_	0.173	0.269	0.090
	p value	0.240	0.064	0.542
**TCRγδ^+^ | gMFI TIM-3**	r_s_	**0.390**	**0.407**	0.268
	p value	**0.007**	**0.005**	0.068
**TCRγδ^+^ | gMFI HLA-DR**	r_s_	**0.332**	0.174	0.138
	p value	**0.023**	0.242	0.355
**TCR Vδ1^+^**	r_s_	0.069	0.219	0.185
	p value	0.639	0.135	0.207
**TCR Vδ1^+^ | gMFI PD1**	r_s_	0.113	0.161	-0.015
	p value	0.442	0.274	0.918
**TCR Vδ1^+^ | gMFI TIM-3**	r_s_	**0.332**	**0.382**	0.238
	p value	**0.023**	**0.008**	0.108
**TCR Vδ1^+^ |gMFI HLA-DR**	r_s_	**0.386**	0.224	0.155
	p value	**0.008**	0.134	0.302
**TCR Vδ2^+^**	r_s_	0.049	-0.003	0.063
	p value	0.741	0.983	0.672
**TCR Vδ2^+^ | gMFI PD1**	r_s_	0.053	-0.029	-0.107
	p value	0.719	0.843	0.468
**TCR Vδ2^+^ | gMFI TIM-3**	r_s_	**0.352**	**0.378**	0.254
	p value	**0.015**	**0.009**	0.084
**TCR Vδ2^+^ |gMFI HLA-DR**	r_s_	0.216	0.056	0.025
	p value	0.145	0.708	0.865
**TCR Vδ1^-^Vδ2^-^**	r_s_	-0.181	**-0.331**	**-0.318**
	p value	0.218	**0.022**	**0.028**
**TCR Vδ1^-^Vδ2^-^ | gMFI PD1**	r_s_	0.212	**0.314**	0.150
	p value	0.148	**0.030**	0.309
**TCR Vδ1^-^Vδ2^-^ | gMFI TIM-3**	r_s_	**0.395**	**0.404**	0.266
	p value	**0.006**	**0.005**	0.071
**TCR Vδ1^-^Vδ2^-^ |gMFI HLA-DR**	r_s_	**0.353**	0.192	0.161
	p value	**0.016**	0.202	0.287

CBMC, Cord blood mononuclear cell; %iRBC Imp, % iRBCs of impression smear (from placenta tissue); Para Per, Peripheral blood parasitemia; Para Pla, Placental blood parasitemia. r_s_, coefficient of correlation; p value, coefficient of significance.

Bold values highlight a p-value less than 0.05.

### Increased HLA-DR expression in CBMC total γδ T cells and Vδ1^+^ and Vδ1^-^Vδ2^-^ subsets during placental malaria

**Figure 4:**
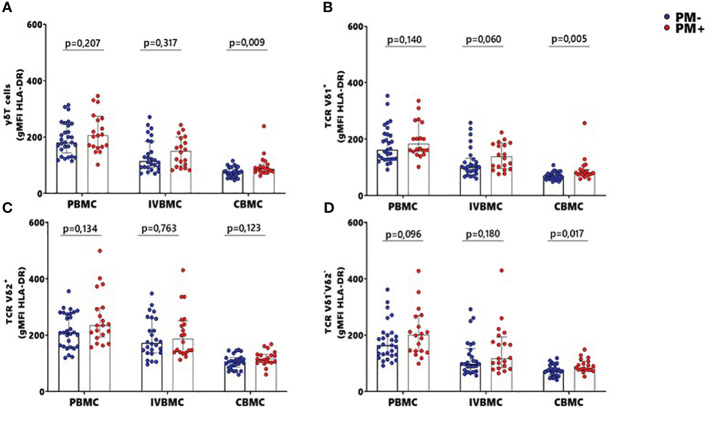
MFI of HLA-DR within γδ T cells and Vδ1^+^, Vδ2^+^, Vδ1^-^Vδ2^-^ subsets in women at delivery. Mean fluorescence intensity (MFI) of HLA-DR within γδ cells and different subsets were analyzed through multiparametric flow cytometry in PBMC, IVBMC and CBMC of PM- (in blue) and PM+ (in red) women. **(A–D)** Mean fluorescence intensity of HLA-DR within **(A)** total TRC γδ^+^ **(B)** TRC Vδ1^+^ **(C)** TRC Vδ2^+^ and **(D)** TRC Vδ1^-^Vδ2 Mean fluorescence intensities were compared in each group using Mann-Whitney test. PM-, Placental Malaria negative women; PM+, Placental Malaria positive women; PBMC, Peripheral blood mononuclear cell; IVBMC, Intervillous blood mononuclear cell; CBMC, Cord blood mononuclear cell. Each dot represents a single individual.

The HLA-DR marker is often used as an activation and/or antigenic presentation phenotype of γδ T cells. In the present study, HLA-DR expression within total γδ T cells, Vδ1^+^ and Vδ1^-^Vδ2^-^ subsets in CBMC were significantly higher in PM+ compared to PM- women (p=0.009, p=0.005 and p=0.017 respectively) (**Figures 4A, B, D**; **Supplementary Figure 2**). Positive correlations were observed between parasitemia from placenta tissue impression smear and HLA-DR expression within total γδ T cells in CBMC (r_s_=0.332; p=0.023), within Vδ1^+^ subset in IVBMC and CBMC (r_s_=0.344, p=0.019 and r_s_=0.386, p=0.008, respectively), and within Vδ1^-^Vδ2^-^ in CBMC (r_s_=0.353; p=0.016) (**Figure 3**; **Tables 3**, **4**).

These data suggest that the absence of direct contact with the parasites lead to expression of HLA-DR within total γδ T cells, Vδ1^+^ and Vδ1^-^Vδ2^-^ subsets.

Similar but non-significant trends in frequencies between PM+ and PM- women were observed for HLA-DR expression within total γδ T cells and its subsets in PBMC and IVBMC (0.060 ≤ p ≤ 0.763) (**Figures 4A, D**). In general, the levels of HLA-DR expression within total γδ T cells and its subsets in PBMC, IVBMC positively correlated with parasitemia from placenta tissue impression smear, placental and peripheral blood (**Figure 3**; **Tables 2**, **3**).

Additionally, HLA-DR expression within total γδ T cells, Vδ2^+^ and Vδ1^-^Vδ2^-^ subsets in PBMC correlated positively with parasitemia from placenta tissue impression smear, placental blood and peripheral blood parasitemia (0.299 ≤ r_s_≤ 0.377; 0.008 ≤ p ≤ 0.037 for total γδ T cells, (0.322 ≤ r_s_ ≤ 0.327, 0.022 ≤ p ≤ 0.024 for Vδ2^+^ and 0.318 ≤ r_s_ ≤ 0.362, 0.011 ≤ p ≤ 0.026 for Vδ1^-^Vδ2). Meanwhile, in the Vδ1^+^ subset, HLA-DR expression correlated with parasitemia only from the placental tissue impression smear and placental blood (0.295 ≤ r_s_≤ 0.330; 0.021 ≤ p ≤ 0.039) (**Figure 3** and **Table 2**). Furthermore, HLA-DR expression within Vδ1^+^ subset in IVBMC correlated positively with placental blood parasitemia (r_s_=0.318; p=0.031). These data are consistent with the hypothesis that the parasites induced T cell activation and/or antigenic peptide presentation by γδ T cells.

### Association of PD1 expression within total γδ T cells, Vδ2^+^ and Vδ1^-^Vδ2^-^ subsets in PBMC and IVBMC with placental malaria

**Figure 5:**
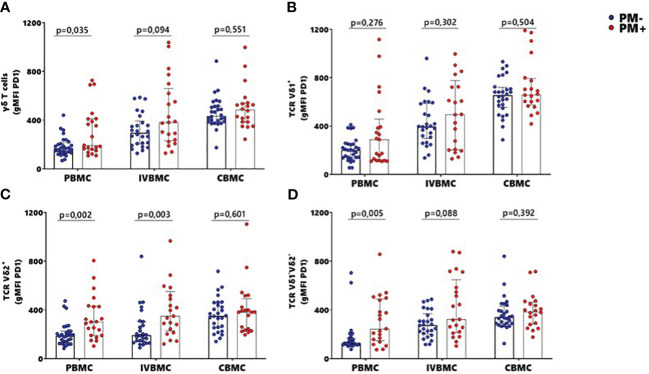
MFI of PD1 within γδ T cells and Vδ1^+^, Vδ2^+^, Vδ1^-^Vδ2^-^ subsets in women at delivery. Mean fluorescence intensity (MFI) of PD1 within γδ T cells and different subsets were analyzed through multiparametric flow cytometry in PBMC, IVBMC and CBMC of PM- (in blue) and PM+ (in red) women. **(A–D)** Mean fluorescence intensity of PD1 within **(A)** total TCR γδ^+^ **(B)** TCR Vδ1^+^ **(C)** TCR Vδ2^+^ and **(D)** TCR Vδ1^-^Vδ2 Mean fluorescence intensities were compared in each group using Mann-Whitney test. PM-, Placental Malaria negative women; PM+, Placental Malaria positive women; PBMC, Peripheral blood mononuclear cell; IVBMC, Intervillous blood mononuclear cell; CBMC, Cord blood mononuclear cell. Each dot represents a single individual.

To further characterize the phenotype of γδ T cells in the context of placental malaria, expression of the exhaustion marker PD1 was compared between PM- and PM+ women (**Figure 5**; **Supplementary Figure 3**). PD1 expression within total γδ T cells and Vδ1^-^Vδ2^-^ subset in PBMC as well as that of Vδ2^+^ subset in PBMC and IVBMC was significantly higher in PM+ women compared to PM- women (p=0.035 and 0.005) and p=0.002 and 0.003, respectively) (**Figures 5AC, D**). Although not significant, PD1 expression within total γδ T cells and Vδ1^-^Vδ2^-^ subset in IVBMC and within Vδ1^+^ subset in PBMC and IVBMC was higher in PM+ compared to PM- women (p=0.094 and 0.088, and p=0.276 and 0.302), respectively) (**Figures 5A, B, D**). No significant difference in PD1 expression between PM+ and PM- women was observed for total γδ T cells, Vδ1^+^, Vδ2^+^ and Vδ1^-^Vδ2^-^ subsets in CBMC (0.392 ≤ p ≤ 0.601) (**Figures 5A–D**). The levels of PD1 expression within total γδ T cells and its subsets in PBMC, IVBMC associated with parasitemia from placenta tissue impression smear, placental and/or peripheral blood (**Figure 3**; **Tables 2**, **3**). Indeed, PD1 expression within total γδ T cells in PBMC correlated positively with parasitemia from placenta tissue impression smear and placental blood parasitemia (r_s_= 0.305; p=0.031 and r_s_= 0.283; p=0.046, respectively) and borderline significance with parasitemia from a placental tissue impression smear in IVBMC (r_s_=0.283; p=0.054) (**Figure 3**; **Tables 2**, **3**). PD1 expression within Vδ2^+^ subset in PBMC and IVBMC correlated positively with parasitemia from placenta tissue impression smear, placental blood and peripheral blood (0.441 ≤ r_s_ ≤ 0.560, 0.00002 ≤ p ≤0.001 and 0.329 ≤ r_s_ ≤ 0.437, 0.002 ≤ p ≤0.026, respectively). Regarding PD1 expression within Vδ1^-^Vδ2^-^ subset, results showed a positive correlation with parasitemia from placenta tissue impression smear in PBMC and IVBMC (r_s_= 0.387, p=0.006 and r_s_= 0.305, p=0.037, respectively). A similar trend was observed with placental blood parasitemia in PBMC (r_s_=0.319, p=0.024), and in CBMC (r_s_=0.314, p=0.030) (**Figure 3**; **Tables 2**–**4**). These data suggest that placental malaria leads to up-regulation of PD1 within total γδ T cells and its subsets.

### Upregulation of TIM3 expression within total γδ T cells, Vδ1^+^, Vδ2^+^ and Vδ1^-^Vδ2^-^ subsets in PBMC, IVBMC and CBMC during placental malaria

**Figure 6:**
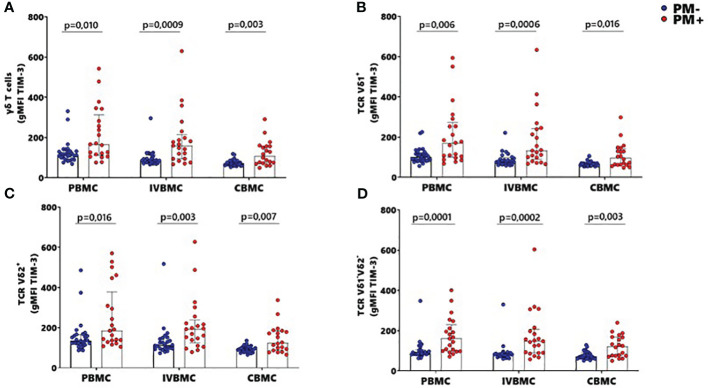
MFI of TIM-3 within γδ T cells and Vδ1^+^, Vδ2^+^, Vδ1^-^Vδ2^-^ subsets in women at delivery. Mean fluorescence intensity (MFI) of TIM-3 γδ T cells and different subsets were analyzed through multiparametric flow cytometry in PBMC, IVBMC and CBMC of PM- (in blue) and PM+ (in red) women. **(A–D)** Mean fluorescence intensity of TIM-3 within **(A)** total TCR γδ^+^ **(B)** TCR Vδ1^+^ **(C)** TCR Vδ2^+^ and **(D)** TCR Vδ1^-^Vδ2 Mean fluorescence intensities were compared in each group using Mann-Whitney test. PM-, Placental Malaria negative women; PM+, Placental Malaria positive women; PBMC, Peripheral blood mononuclear cell; IVBMC, Intervillous blood mononuclear cell; CBMC, Cord blood mononuclear cell. Each dot represents a single individual.

The activation and function of γδ T cells can be regulated by the expression of immunoregulatory markers. In this study, expression of the exhaustion marker TIM-3 within total γδ T cells, Vδ1^+^, Vδ2^+^ and Vδ1^-^Vδ2^-^ subsets in PBMC, IVBMC and CBMC was significantly higher in PM+ compared to PM- women (0.0001 ≤ p ≤ 0.016) (**Figures 6A–D**; **Supplementary Figure 4**). As presented in **Figure 3** and **Tables 2**–**4**, TIM-3 expression within total γδ T cells in PBMC, IVBMC, and CBMC positively correlated with different parasitemia from placenta tissue impression smear, placental blood and peripheral blood parasitemia (0.408 ≤ r_s_ ≤ 0.522, 0.0002 ≤ p ≤ 0.007). Similar trends were observed for its two subsets (Vδ1^+^, Vδ2^+^) in IVBMC (0.318 ≤ r_s_ ≤ 0.347, 0.017 ≤ p ≤ 0.034 for Vδ1^+^ subset and 0.395 ≤ r_s_ ≤ 0.541, 0.0001 ≤ p ≤ 0.007 for Vδ2^+^ subset), and for Vδ1^-^Vδ2^-^ subset in PBMC and IVBMC (0.435 ≤ r_s_ ≤ 0.561, 0.00004 ≤ p ≤ 0.003). TIM-3 expression on the two subsets (Vδ1^+^ and Vδ2) also correlated positively with parasitemia from placenta tissue impression smear and placental blood parasitemia in PBMC and CBMC (0.332 ≤ r_s_ ≤ 0.394, 0.005 ≤ p ≤ 0.023) and in CBMC for Vδ1^-^Vδ2^-^ subset (r_s_= 0.395, p= 0.006 and r_s_= 0.404; p= 0.005; respectively) (**Figure 3** and **Tables 2**–**4**). Together, these data indicate that placental malaria leads to increased expression of the TIM-3 immunoregulatory markers within total γδ T cells.

### Association of γδ T cells phenotype in PBMC, IVBMC and with maternal hemoglobin level, birth weight, and fetal hemoglobin level

**Figure 7:**
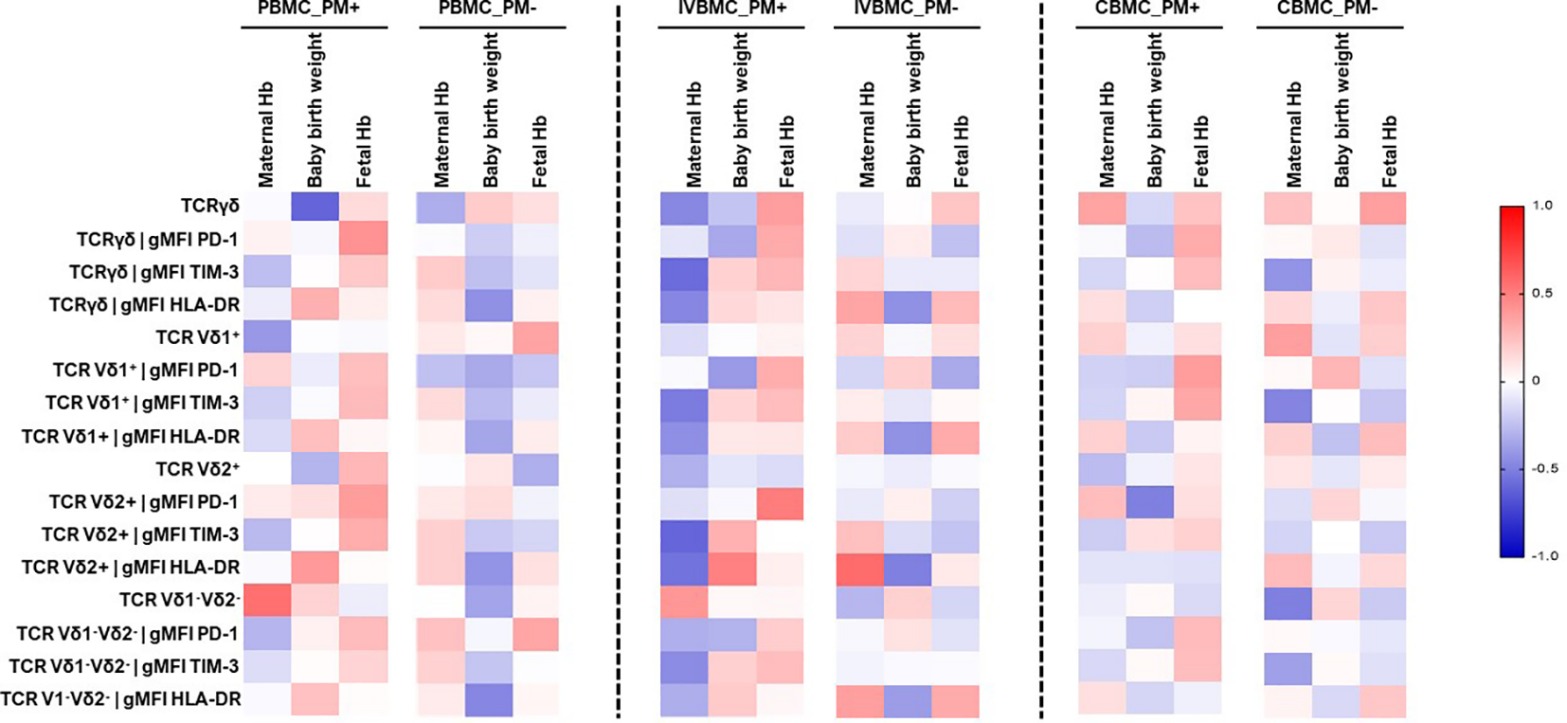
Heatmap representing the correlation between the γδ T cells phenotype and pregnancy outcomes. The correlation between γδ T cells profile in PBMC, IVBMC, CBMC and pregnancy outcomes was determined using Sperman’s rank Order correlation test. Hb, Hemoglobin level; PM-, Placental Malaria negative women; PM+, Placental Malaria positive women; PBMC, Peripheral blood mononuclear cell; IVBMC, Intervillous blood mononuclear cell; CBMC, Cord blood mononuclear cell.

In the present study, we investigated the relationship between pregnancy outcomes and the γδ T cells phenotype in PBMC, IVBMC and CBMC (**Figure 7**; **Supplementary Tables 1**–**3**). In PM+ women, maternal hemoglobin levels correlated negatively with the frequency of total γδ T cells (r_s_=-0.468; p=0.032), and with expression levels of the exhaustion marker TIM-3 within total γδ T cells, Vδ1^+^, Vδ2^+^, Vδ1^-^Vδ2^-^ subsets in IVBMC (-0.601 ≤ r_s_ ≤ -0.452, 0.004 ≤ p ≤ 0.040). In addition, maternal hemoglobin levels correlated negatively with expression of the activation marker HLA-DR within total γδ T cells and Vδ2^+^ subset (r_s_=-0.473, p=0.035 and r_s_=-0.550, p=0.012, respectively) (**Figure 7**; **Supplementary Table 2**). In contrast, maternal hemoglobin levels correlated positively with the frequency of Vδ1^-^Vδ2^-^ subsets in PBMC (r_s_=0.560, p=0.008). Fetal hemoglobin levels correlated positively with PD1 expression within total γδ T cells in PBMC and within Vδ2^+^ subset in IVBMC (r_s_=0.507, p=0.019) (**Figure 7**; **Supplementary Tables 1**, **2**). Regarding birth weight, a negative correlation was observed with the frequency of total γδ T cells in PBMC (r_s_=-0.606; p=0.006) and the PD1 expression within Vδ2^+^ subset in CBMC (r_s_=-0.501, p=0.034). On the other hand, HLA-DR expression within Vδ2^+^ subset in IVBMC correlated positively with birth weight (r_s_=0.489, p=0.040) (**Figure 7**; **Supplementary Tables 1**, **3**). Together, these results suggest that in placental malaria, the expansion of total γδ T cells as well as its subsets and the expression of the activation and exhaustion markers may be associated with poor pregnancy outcomes or newborn protection.

### Variation of phenotypic profile of γδ T cells with maternal age, gestational age and parity

**Figure 8:**
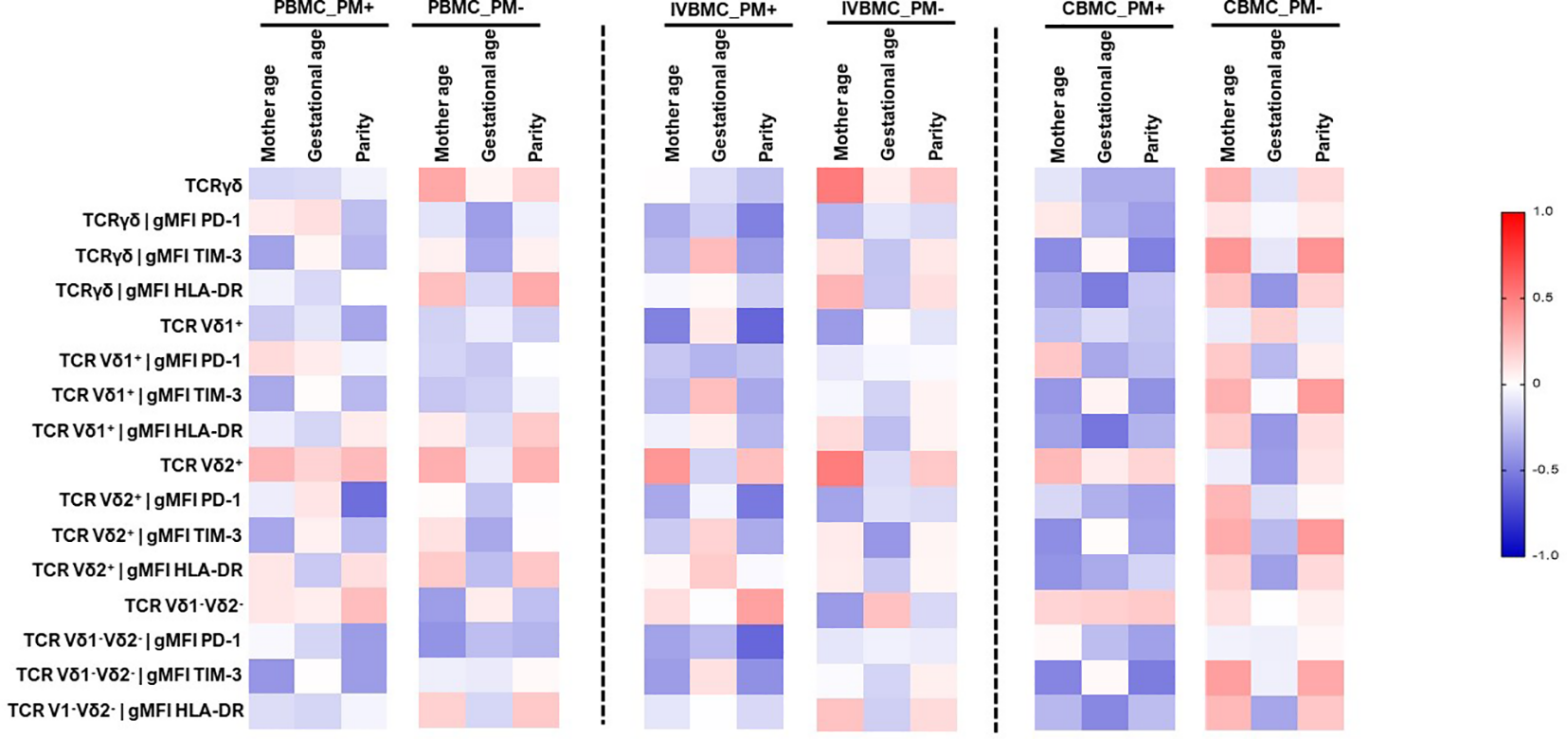
Heatmap representing the correlation between the γδ T cells phenotype and mother age, gestational age, parity. The correlation between γδ T cells profile in PBMC, IVBMC, CBMC and gestational age, parity was determined using Sperman’s rank Order correlation test. PM-, Placental Malaria negative women; PM+, Placental Malaria positive women; PBMC, Peripheral blood mononuclear cell; IVBMC, Intervillous blood mononuclear cell; CBMC, Cord blood mononuclear cell.

Cell profile is often associated with maternal age, parity and gestational age. In this study, we examined the relationship between these parameters and the γδ T cells phenotype in PBMC, IVBMC and CBMC (**Figure 8**; **Supplementary Tables 4**–**6**). In PM+, maternal age correlated negatively with TIM-3 expression within total γδ T cells and Vδ1^-^Vδ2^-^ subset in CBMC (r_s_=-0.454, p=0.045 and r_s_=-0.477, p=0.034, respectively]. HLA-DR expression within total γδ T cells, Vδ1^+^, and Vδ1^-^Vδ2^-^ subsets in CBMC correlated negatively with gestational age (r_s_=-0.513, p=0.025, r_s_=-0.541, p=0.017 and r_s_=-0.470, p=0.042, respectively) (**Figure 8**; **Supplementary Table 6**).

Regarding parity, the frequency of Vδ1^+^ subset in IVBMC correlated negatively with parity (r_s_=-0.603, p=0.005). PD1 expression within Vδ2^+^ subset in PBMC and IVBMC as well as within total γδ T cells and Vδ1^-^Vδ2^-^ subsets in IVBMC correlated negatively with parity (-0.598 ≤ r_s_ ≤ -0.498, 0.004 ≤ p ≤ 0.022). In addition, TIM-3 expression within total γδ T cells and Vδ1^-^Vδ2^-^ subset correlated negatively with parity (r_s_=-0.497, p=0.026 and r_s_=-0.513, p=0.021, respectively). The data suggest that expansion of γδ T cells may be affected by some of the above studied parameters.

## Discussion

γδ T cells are unconventional T cells that contribute significantly to global immunity during malaria.

Despite recent efforts that have demonstrated dichotomous functions of γδ T cells during *Plasmodium falciparum* infections in children living in malaria endemic areas (15, 31), there is lack of data regarding the role of these cells in placental malaria. Understanding the role of γδ T cells in the pathophysiology of placental malaria could lead to the development of innovative therapies and vaccines. This study aimed to investigate the involvement of γδ T cells and its subsets in *Plasmodium falciparum* placental malaria and pregnancy outcomes in women at delivery in Yaoundé, Cameroon.

Our data show variability in the distribution of γδ T cells and its subsets as a function of blood compartment (PBMC, IVBMC and CBMC) and a differential impact of placental *P. falciparum infection* on each compartmental cell subset profile. Such variabilities are likely to define the magnitude of immune responses in each compartment and the impact on the pregnant mother or the developing fetus. In fact, a high frequency of total γδ T cells, Vδ1^+^ subset, and a reduced frequency of the Vδ2^+^ subset was observed in peripheral and placental blood from PM+ women. This variation in the frequency of Vδ1^+^ and Vδ2^+^ subsets suggest an adaptation of γδ T lymphocytes in response to placental malaria infection. These findings are consistent with observations in children showing increased frequency of the Vδ1^+^ subset and decreased frequency of the Vδ2^+^ subset with repeated infection (15, 32). Repeated infections in children were characterized by waves of clonal selection in the TCR Vδ1^+^ repertoire, coupling with the differentiation of the naive Vδ1^+^ subset into cytotoxic effector Vδ1^+^ subset (32). For the first time, we show that expression of the activation marker HLA-DR within total γδ T cells, Vδ1^+^ and Vδ1^-^Vδ2^-^ subsets were higher in CBMC from PM+ women compared to that from PM- women. These results are consistent with those of Howard et al. (33), who showed increased expression of antigen-presenting cell-associated markers such as HLA-DR in patients infected with *Plasmodium falciparum*. Compared with CBMC, we observed significant heterogeneity in HLA-DR expression in PBMC and IVBMC, presumably resulting from direct activation by circulating parasites or their products (34, 35).

Chronic activation of T cells may result in exhaustion. The present study observed an association between *Plasmodium falciparum* placental malaria and expression of the exhaustion marker TIM-3 in all three blood compartments. These observations suggest that TIM-3 expression may control γδ T cell functions during malaria. In fact, in children living in a context of high or continuous malaria transmission, an increase in the expression of Tim-3 as well as the immunoregulatory gene HAVCR2 (encoding the inhibitory Tim-3 receptor) on Vδ2^+^ subset was observed and associated with decreased production of pro-inflammatory cytokines (31, 36). TIM-3 expression can be regulated by IL-12 and IL-18 (36), and IL-12 may be produced by several immune cells, including monocytes in the placenta of infected women (37).

Increasing evidence show the involvement of γδ T cells in the pathophysiology of malaria. This study demonstrates for the first time the association between the frequency of total γδ T cells and subsets, the expression of exhaustion and activation markers within these cells in PM+ women and pregnancy outcomes. In fact, TIM-3 expression within total γδ T cells and the Vδ1^+^, Vδ2^+^ and Vδ1^-^Vδ2^-^ subsets in IVBMC, and HLA-DR expression within total γδ T cells and the Vδ2^+^ subset in IVBMC and within the Vδ1^+^ subset in CBMC negatively associated with maternal hemoglobin levels. Additionally, the frequency of total γδ T cells in PBMC and the expression of PD1 within Vδ2^+^ subset in CBMC also negatively associated with birth weight. In contrast, the frequency of Vδ1^-^Vδ2^-^ subset in PBMC and HLA-DR expression within Vδ2^+^ subset in IVBMC positively associated with maternal hemoglobin level and birth weight, respectively. Although the mechanisms are not fully understood, activated γδ T cells have been shown to acquire cytolytic potentials by upregulating IFN-γ and various cytotoxic effector proteins (38). IFN-γ, although implicated in protection against placental malaria, could promote low birth weight and low maternal hemoglobin levels (39, 40). However, further studies are needed in the context of placental malaria. Similar outcomes were observed in a mouse model where high proportions of Vγ9Vδ2 T cells were associated with increased plasma levels of pro-inflammatory cytokines, notably TNF alpha and IFNγ, leading to cerebral malaria (41, 42). These findings are consistent with observations in acute myeloid leukemia and myelodysplastic syndromes, where high TIM-3 expression has been associated with pathogenesis and disease progression (43). However, our results contrast with those obtained in children that show a decrease in clinical signs with TIM-3 expression on γδ T cells (31). Such differences may result from specific *in situ* immune responses occurring in the placenta (44).

Another major complication of placental malaria is fetal anemia, which is a risk factor for infant mortality. Interestingly, our study revealed for the first time a positive correlation between fetal hemoglobin levels and PD1 expression within total γδ T cells in PBMC and within Vδ2^+^ subset in IVBMC. This suggests that γδ T cells may play a protective role in limiting fetal anemia during placental malaria. In fact, Hsu et al. showed that prolonged PD1 expression on neonatal Vδ2 lymphocytes inhibits TNF-α production and granule mobilization (44, 45). Although the role of IL-17 is still poorly understood, some studies highlight IL-17 as a regulatory cytokine that is essential for maintaining a successful pregnancy (45, 46). In general, the function of γδ T cells in *Plasmodium falciparum* placental malaria would depend on the compartments involved and phenotypes. This study also demonstrates an association between PD1 expression within total γδ T cells, its subsets and parity. In fact, PD1 expression within Vδ2^+^ subset in PBMC, and within total γδ T cells, Vδ1^+^, Vδ2^+^ and Vδ1^-^Vδ2^-^ subsets in IVBMC of PM+ women decreased with increasing parity. A similar trend was observed between TIM-3 expression within total γδ T cells, Vδ1^-^Vδ2^-^ subset in CBMC of PM+ women and parity. A negative correlation was observed between HLA-DR expression within total γδ T cells, Vδ1^+^ and Vδ1^-^Vδ2^-^ subsets in CBMC of PM+ women and gestational age. Although the mechanisms remain unclear, these results suggest that HLA-DR expression within γδ T cells and their subsets in CBMC may affect pregnancy outcomes. Indeed, successful pregnancy is characterized by complete absence of HLA-I and HLA-II expression in the placental syncytiotrophoblast (47). In addition, other studies have shown high expression of circulating HLA-DR in syncytiotrophoblast-derived extracellular vesicles (STBEV) in women with pre-eclampsia compared with normal pregnant women (48, 49).

Our results are of paramount importance for the understanding of malaria immunity and highlight the essential role of γδ T cells in the immune response during placental malaria. Moreover, a better understanding of these immune mechanisms will open the way to possible immunotherapeutic research orientations against placental malaria. A limitation of this study is the lack of data on the functional response of γδ T cells in the context of placental malaria. Larger sample sizes are essential to better explain the heterogeneity observed in cell populations and reinforce correlations with poor pregnancy outcomes. Deciphering the repertoire of γδ T cell responses is relevant for understanding their role in the immunopathophysiology of placental malaria and pregnancy outcomes.

## Conclusion

Taken together, the data suggest a role for γδ T cells in host immune responses to *P. falciparum* infection during pregnancy, with the Vδ1^+^ and Vδ2^+^ subsets exhibiting opposing associations with pregnancy outcomes such as gestational age, maternal hemoglobin levels and birth weight. These findings are crucial for our understanding of the immunopathogenic mechanisms of PM, with relevance for the development of new therapeutic strategies against placental malaria.

## Data availability statement

The original contributions presented in the study are included in the article/**Supplementary Material**. Further inquiries can be directed to the corresponding author.

## Ethics statement

The studies involving humans were approved by National Ethics Committee of Cameroon (Ethical Clearances No 2021/02/1331/CE/CNERSH/SP). Administrative authorizations were obtained from the Cameroon Ministry of Public Health (No 631-1221 of 28th April 2021, D30-459/L/MINSANTE/SG/DROS). The studies were conducted in accordance with the local legislation and institutional requirements. Written informed consent for participation in this study was provided by the participants’ legal guardians/next of kin.

## Author contributions

CN: Conceptualization, Data curation, Formal analysis, Investigation, Methodology, Software, Writing – original draft. BT: Data curation, Investigation, Methodology, Writing – original draft. BB: Data curation, Investigation, Methodology, Writing – original draft. BF: Investigation, Software, Writing – original draft. BZ: Investigation, Methodology, Writing – original draft. RS’O: Investigation, Methodology, Writing – original draft. BN: Investigation, Methodology, Writing – original draft. RL: Supervision, Writing – review & editing. JD: Writing – review & editing. RA: Writing – review & editing. LA: Validation, Writing – review & editing. RM: Conceptualization, Data curation, Formal analysis, Funding acquisition, Resources, Supervision, Validation, Writing – original draft, Writing – review & editing.
