# Supplementary figures and images for: Phenotypic changes of γδ T cells in Plasmodium falciparum placental malaria and pregnancy outcomes in women at delivery in Cameroon
Source: Front Immunol. 2024 May 17;15:1385380. doi: 10.3389/fimmu.2024.1385380 (PMC11140112; doi:10.3389/fimmu.2024.1385380)

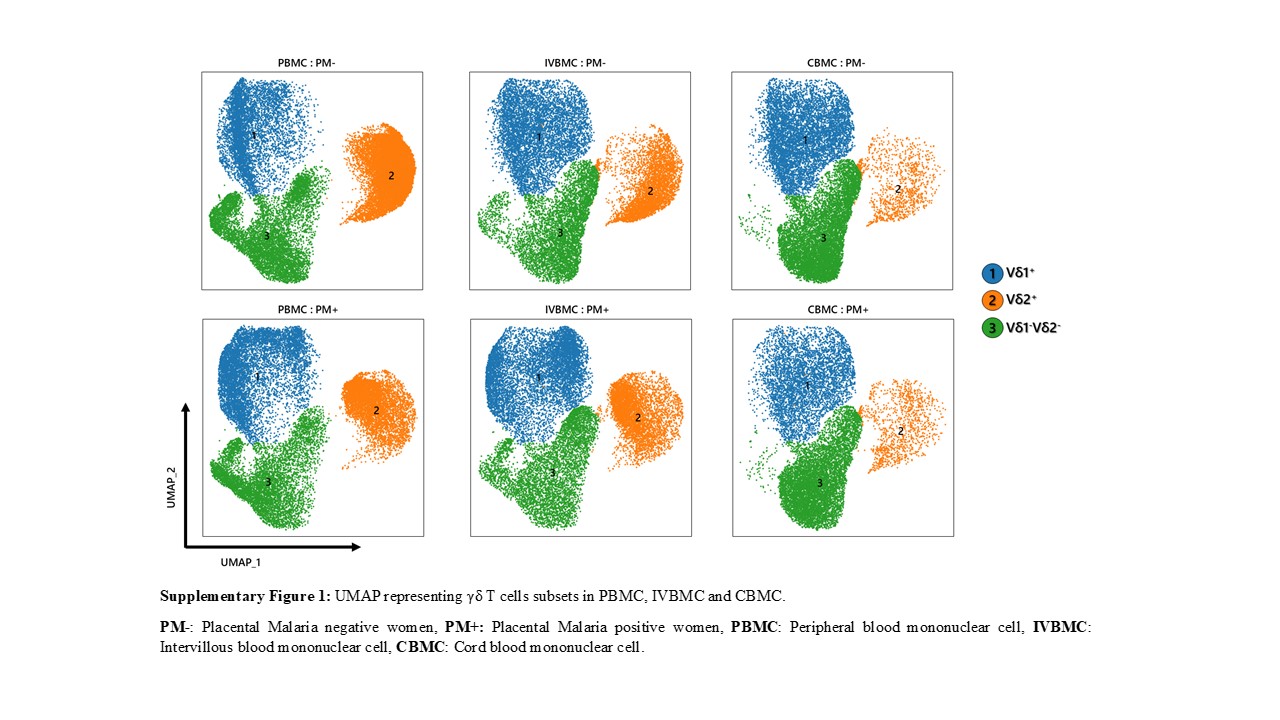

Supplement: Supplementary file 1 [file Image_1.jpeg]

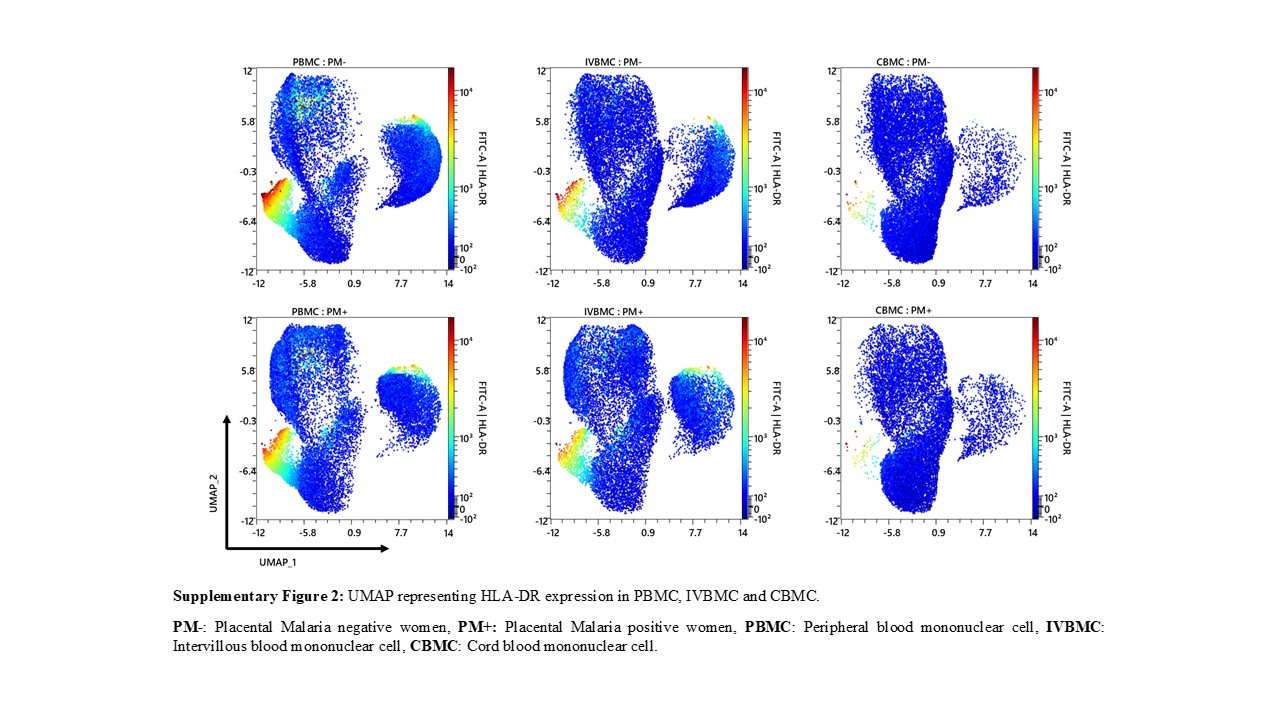

Supplement: Supplementary file 2 [file Image_2.jpeg]

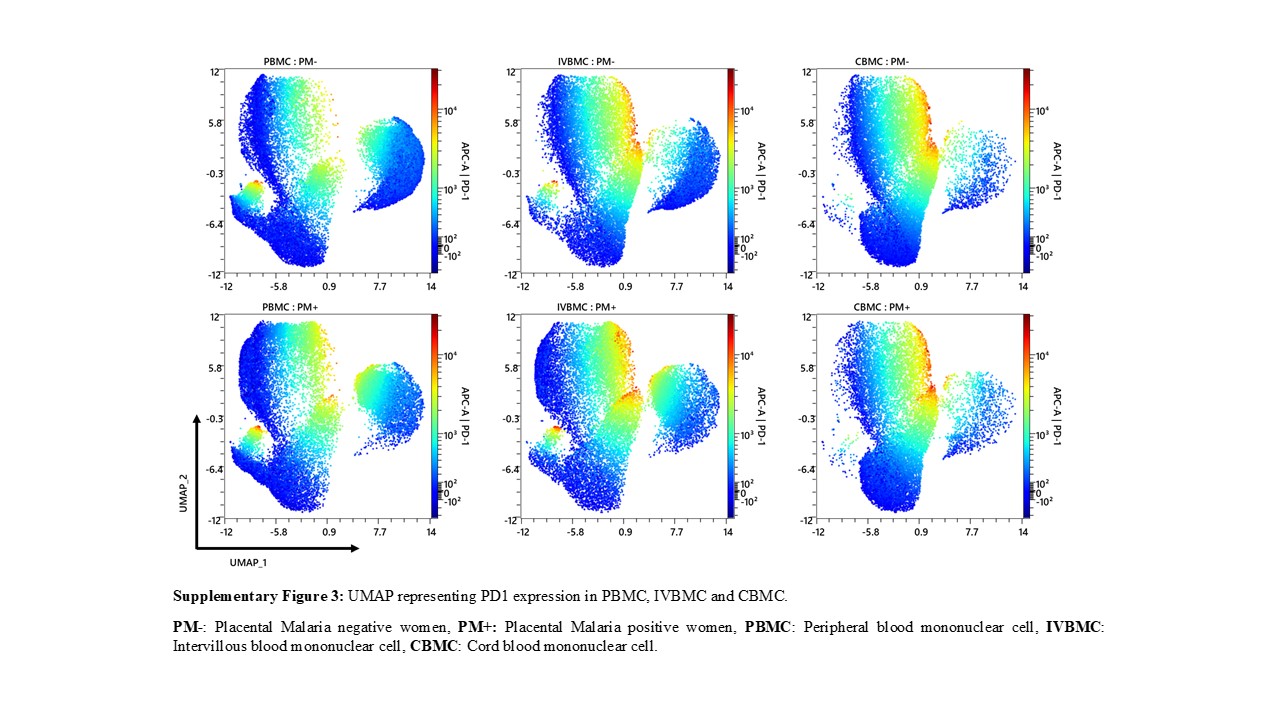

Supplement: Supplementary file 3 [file Image_3.jpeg]

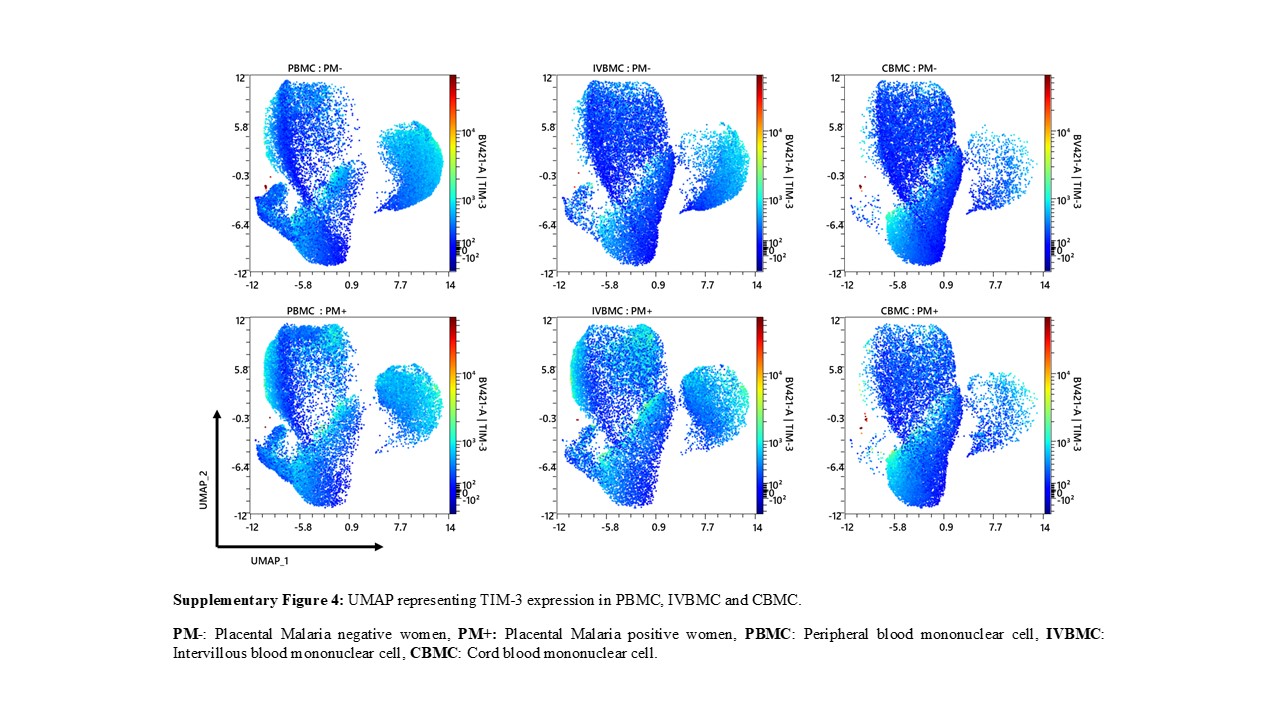

Supplement: Supplementary file 4 [file Image_4.jpeg]

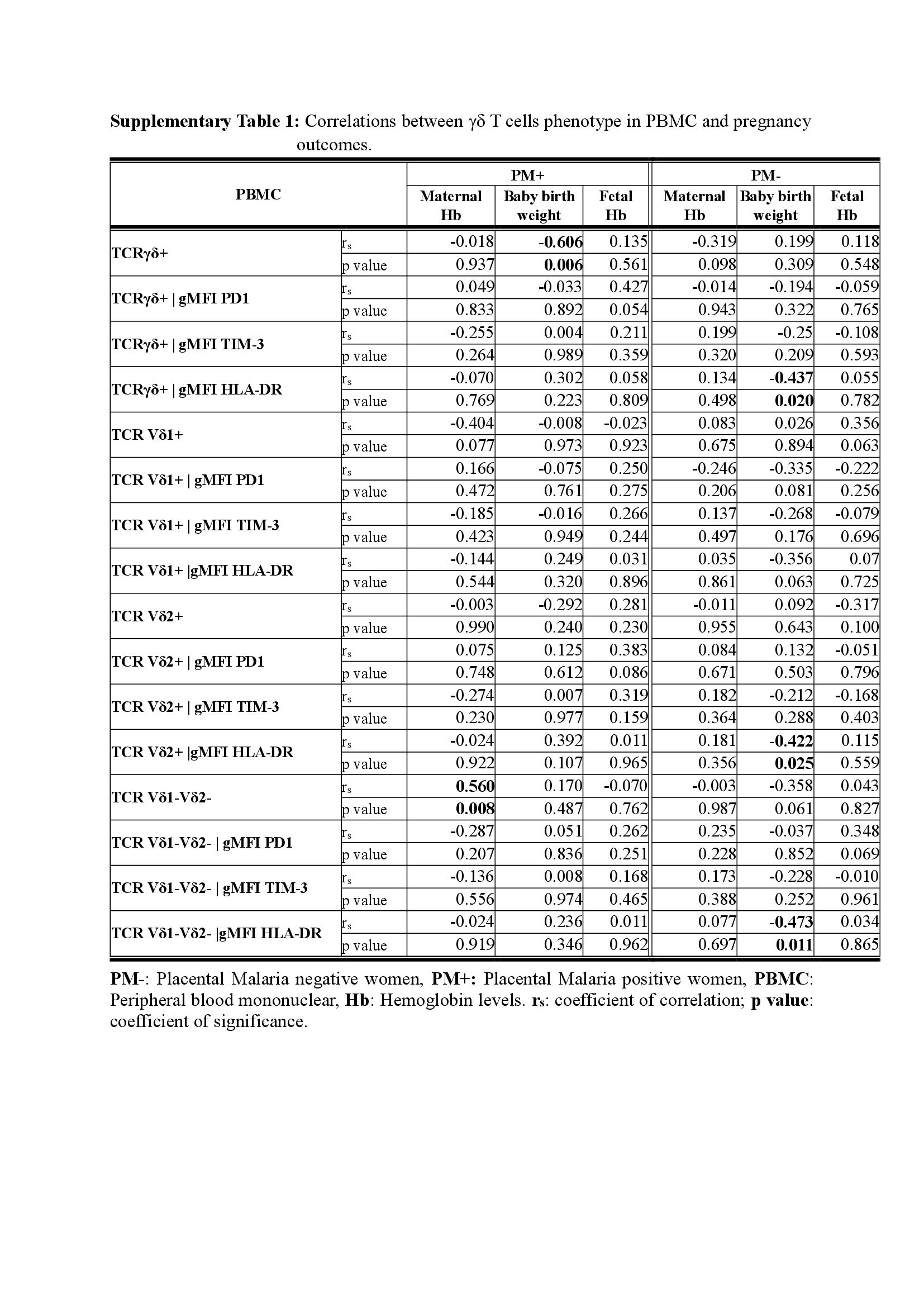

Supplement: Supplementary file 5 [file Image_5.jpeg]

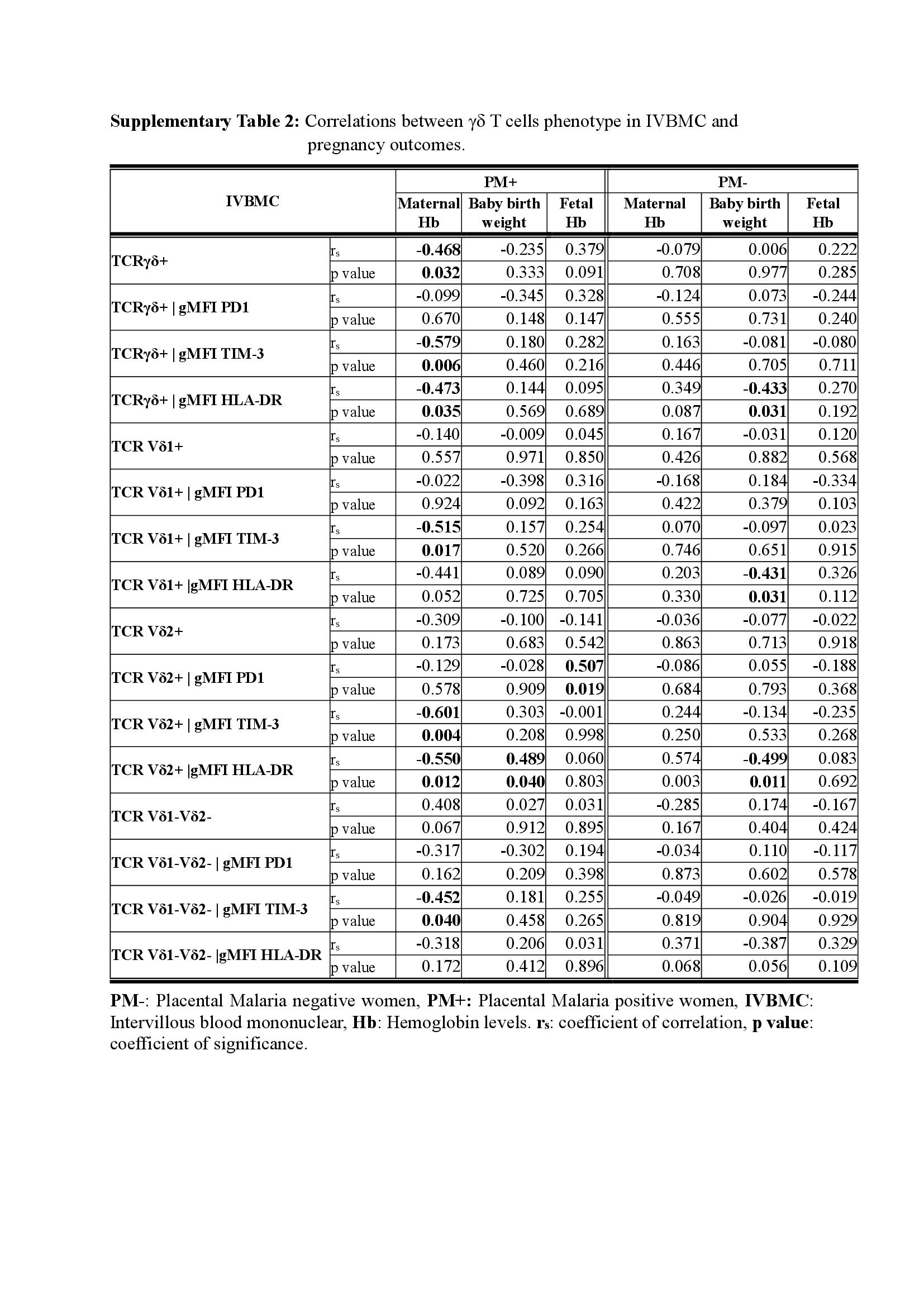

Supplement: Supplementary file 6 [file Image_6.jpeg]

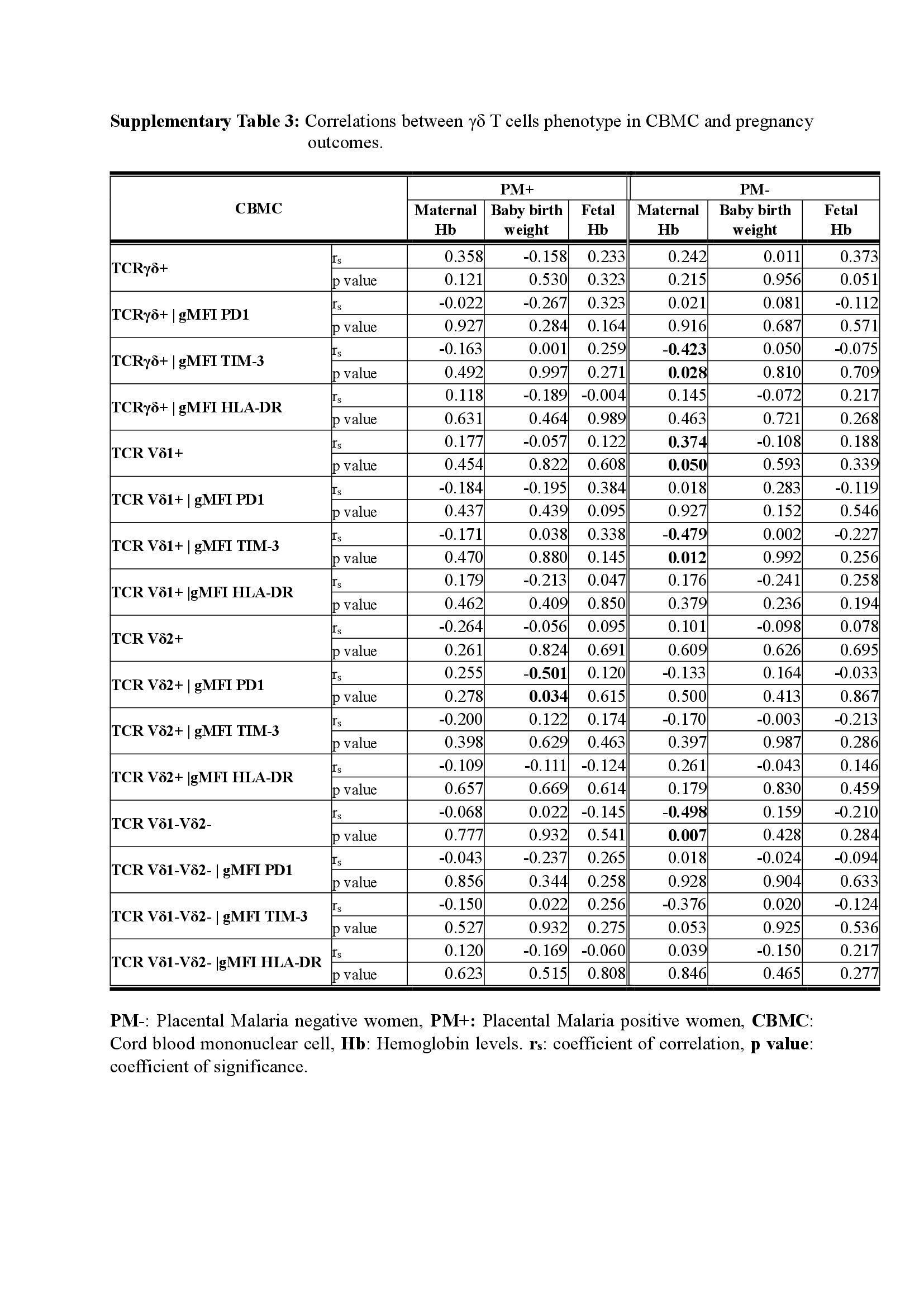

Supplement: Supplementary file 7 [file Image_7.jpeg]

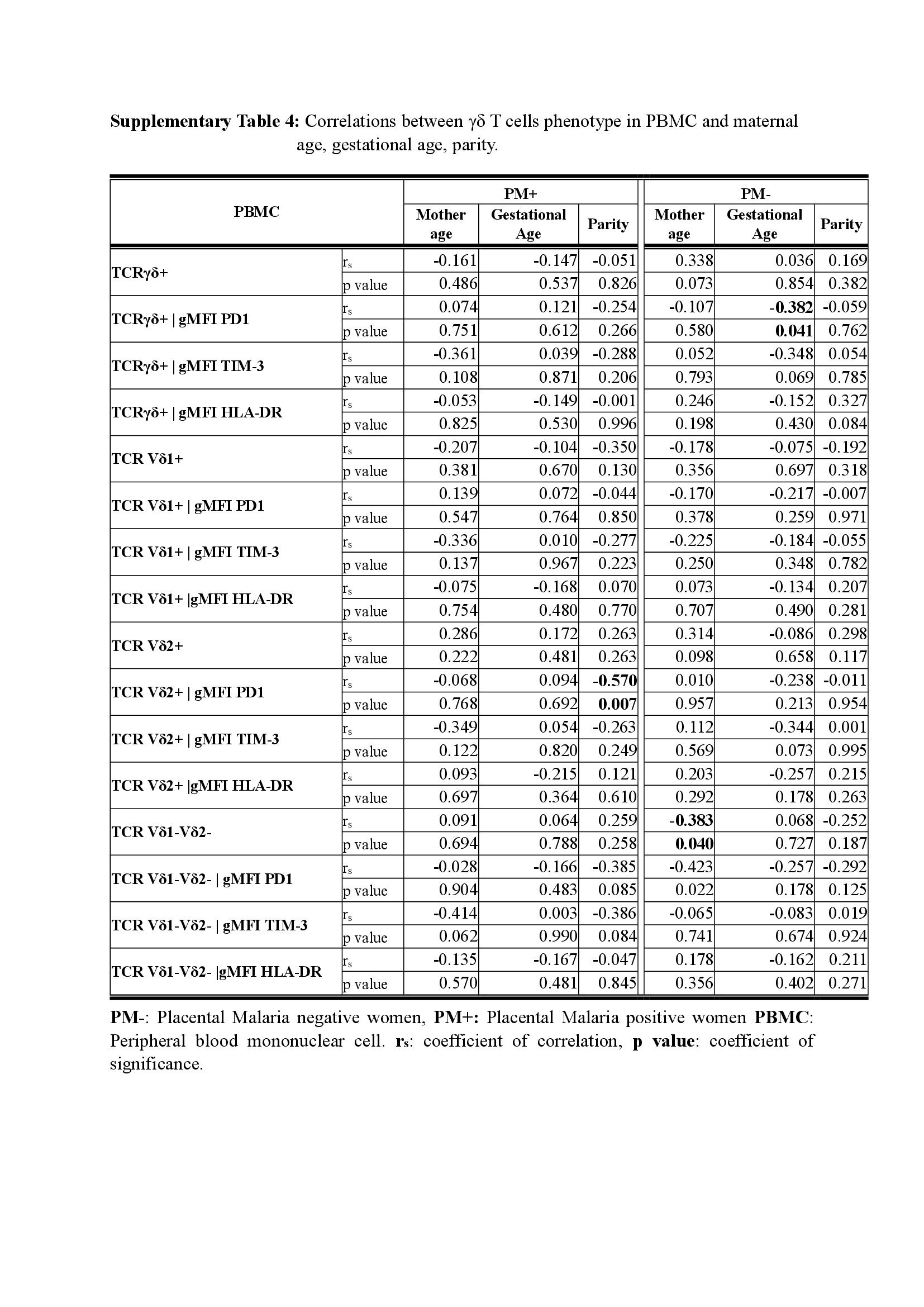

Supplement: Supplementary file 8 [file Image_8.jpeg]

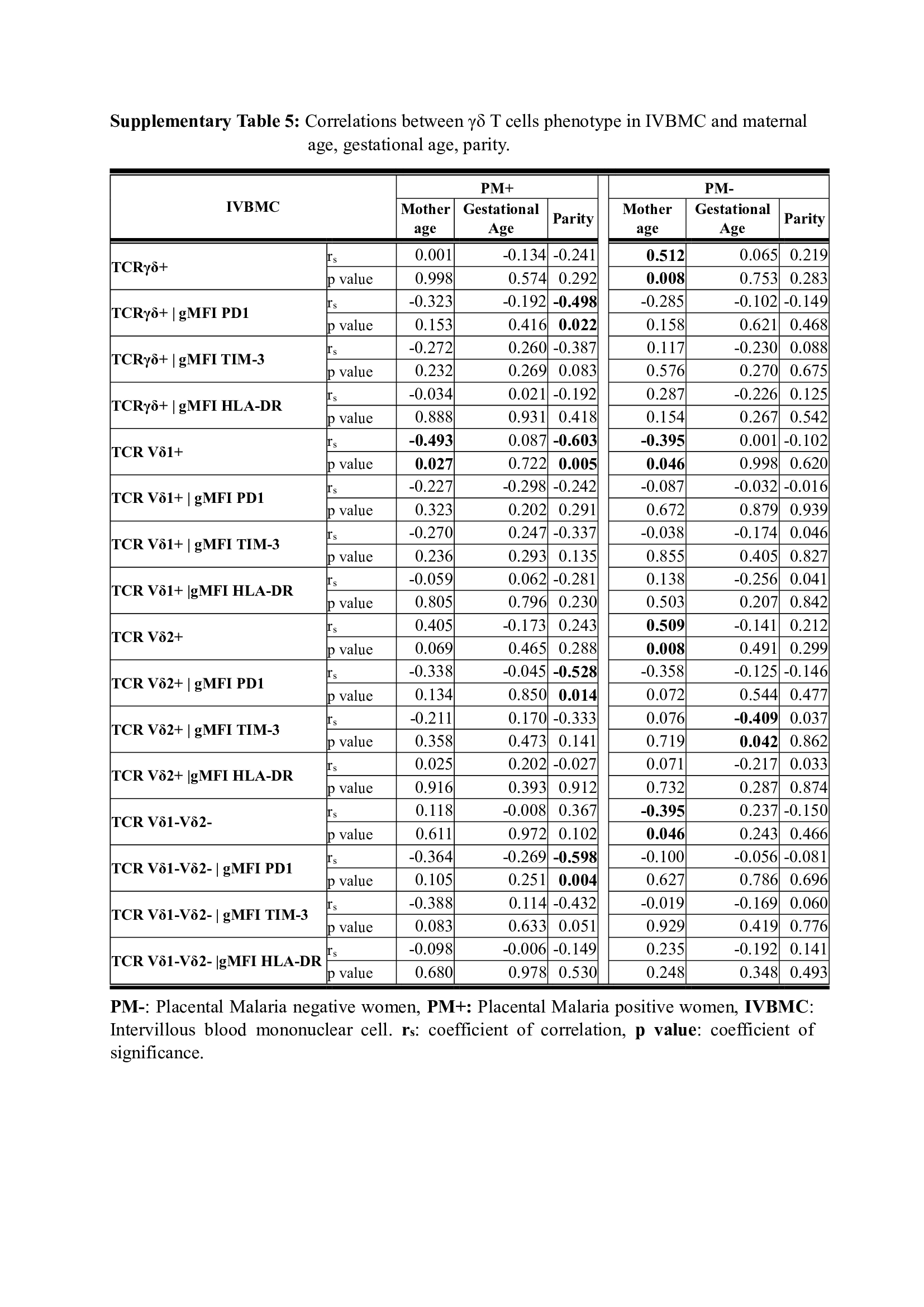

Supplement: Supplementary file 9 [file Image_9.jpeg]

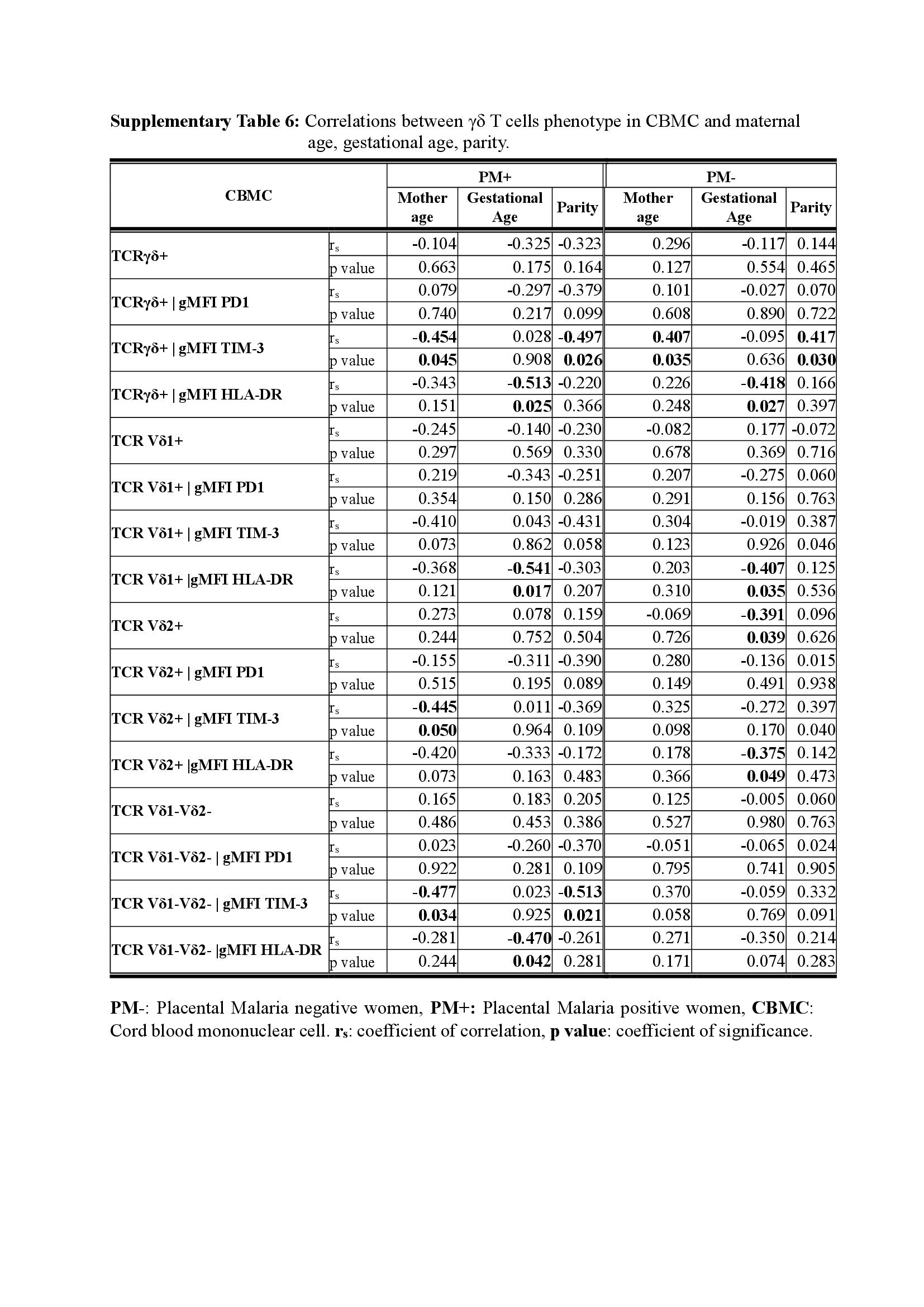

Supplement: Supplementary file 10 [file Image_10.jpeg]
